# Seed selection and storage with nano-silver and copper as potential antibacterial agents for the seagrass *Zostera marina*: implications for habitat restoration

**DOI:** 10.1038/s41598-019-56376-0

**Published:** 2019-12-27

**Authors:** Shaochun Xu, Yi Zhou, Shuai Xu, Ruiting Gu, Shidong Yue, Yu Zhang, Xiaomei Zhang

**Affiliations:** 1 CAS Key Laboratory of Marine Ecology and Environmental Sciences, Institute of Oceanology, Chinese Academy of Sciences, Qingdao, 266071 China; 2 Laboratory for Marine Ecology and Environmental Science, Qingdao National Laboratory for Marine Science and Technology, Qingdao, 266237 China; 3 University of Chinese Academy of Sciences, Beijing, 100049 China; 4 Center for Ocean Mega-Science, Chinese Academy of Sciences, Qingdao, 266071 China

**Keywords:** Restoration ecology, Plant sciences

## Abstract

Globally, seagrass meadows are extremely important marine ecosystems that are disappearing at an alarming rate. Therefore, research into seagrass restoration has become increasingly important. Various strategies have been used in *Zostera marina* L. (eelgrass) restoration, including planting seeds. To improve the efficiency of restoration by planting seeds, it is necessary to select high-quality seeds. In addition, a suitable antibacterial agent is necessary for wet storage of desiccation sensitive seeds to reduce or inhibit microorganism infection and seed decay. In the present study, an efficient method for selecting for high-quality eelgrass seeds using different specific gravities of salt water was developed, and potential antibacterial agents (nano-silver and copper sulfate) for seed storage were assessed. The results showed that the highest proportion of intact seeds (72.91 ± 0.50%) was recorded at specific gravities greater than 1.20. Therefore, specific gravities greater than 1.20 can be used for selecting high-quality eelgrass seeds. During seed storage at 0 °C, the proportion of intact seeds after storage with nano-silver agent was over 90%, and also higher than 80% with copper sulfate agent, which was significantly higher than control treatments. The findings revealed a potential selection method for high-quality seeds and long-term seed storage conditions for *Z. marina*, which could facilitate conservation and habitat restoration.

## Introduction

Seagrasses are important habitat-forming angiosperms in global coastal marine ecosystems^1^. Seagrass communities stabilize sediment, alter water flow, provide habitat, food, and nursery areas for a variety of marine organisms^2–7^, as well as reducing exposure to bacterial pathogens of humans, fishes, and invertebrates^8^. Seagrass meadows also serve as key sites for global carbon storage in the biosphere^9–11^. However, because of natural and anthropogenic disturbances, seagrasses are declining worldwide at an alarming rate, with little recovery^7,12–16^. The importance of seagrasses to communities and ecosystems worldwide is now recognized, and global conservation, management, and restoration of seagrass beds has become increasingly important in recent decades^4,12,17–24^.

As an iconic seagrass species, the eelgrass *Zostera marina* L. is a representative member of the Zosteraceae, and is distributed circum-globally throughout the Northern Hemisphere^1,25^. In China, eelgrass is distributed in the coastal areas of Liaoning to Shandong Province^26^. In recent years, there has been a marked decline (>80%) in the distribution of eelgrass meadows in inshore areas of Shandong Province, and some eelgrass meadows have disappeared since the 1980s^27^. Therefore, eelgrass restoration has become increasingly important in recent decades. Various strategies have been used to restore seagrass beds^17,20,28^, including transplanting adult *Z. marina* shoots^29–31^, and planting seeds^32–36^.

Seagrass seed banks generally include germinated seeds, rotten seeds and intact seeds. Intact seeds should be selected for indoor seed research (including seed storage and germination) and field seed restoration. However, selecting large amounts of seagrass seeds is labor intensive and expensive. For the seeds of terrestrial plants, the buoyancy of salt water can be utilized to separate grass seeds, impurities and empty seeds. This separation method improves seed quality, increases germination rates, and reduces disease occurrence^37–43^. However, little is known about the use of salt selection for the seeds of marine plants. In the present study, different specific gravities (SGs) of salt water were used to select high-quality eelgrass seeds.

Seed desiccation tolerance is generally divided into three broad categories^44^: desiccation tolerant (orthodox), intermediate, and desiccation sensitive (recalcitrant). Desiccation sensitive seeds cannot be stored using traditional seed banking techniques (dry preservation). Wyse and Dickie (2017) predicted that most flowering plants including Alismatales produce desiccation-tolerant seeds globally^45^. However, the majority of seagrass seeds are suggested to be desiccation sensitive, although little is known about the seed ecology of these species. Short-term drying experiments have shown that *Z. marina* L. seeds are strongly desiccation sensitive, losing their viability completely after a 24 h desiccation period^46^. To date, only *Ruppia* species such as *R. maritima* and *R. sinensis* have been identified as being intermediate in terms of seed desiccation tolerance, and their seeds can survive dry conditions for a number of months^47,48^. Therefore, the majority of seagrass seeds must be stored in seawater. According to our recent research on long-term seed storage, 0 °C and salinity 40–50 psu are suitable conditions for eelgrass seed storage (unpublished data). However, regular water exchange operations are required to reduce microorganism infections, which can increase seed losses. To reduce or inhibit microorganism infections and seed decay, two antibacterial agents, nano-silver and copper sulfate, at different concentration levels, were utilized. The aims of the study were to identify the optimal SG of salt water for the selection of high-quality eelgrass seeds, and appropriate antibacterial agents for seed storage, which could facilitate the restoration of *Z. marina* meadows via seeds.

## Results

### Seed selection using different specific gravities of salt water

**Figure 1:**
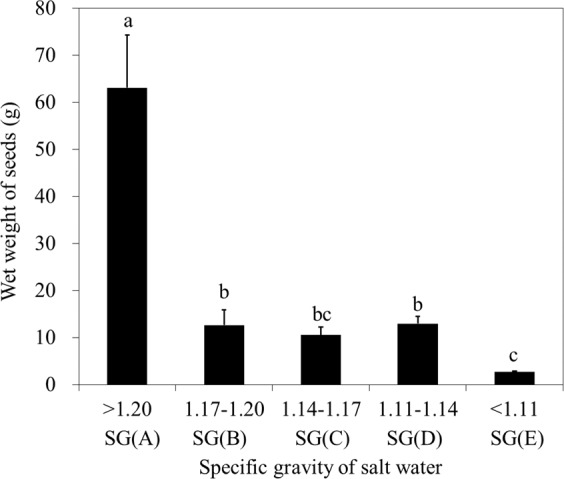
Wet weight of eelgrass seeds at different SGs of salt water. The wet weight of selected seeds differed significantly (Kruskal-Wallis test, χ2 = 11.855, n = 15, *P* = 0.018) at different SGs. The wet weight of SG (A) was significantly higher than that of the other classes [SG (B–E)] (two sided t-test, *P* < 0.02 for each SG). Different letters indicate significant differences at *P* < 0.05 (mean ± SD). Bars represent sd (n = 3).

The wet weight of selected seeds differed significantly (Kruskal-Wallis test, χ2 = 11.855, n = 15, *P* = 0.018) at different SGs (Fig. 1). The wet weight of SG (A) accounted for a large proportion (61.93%) of the total weight of selected seeds. Therefore, the wet weight (50.17–70.54 g) of SG (A) was significantly higher than that of the other classes [SG (B–E)] (two sided t-test, *P* < 0.02 for each SG). There was no significant difference in wet weight among SG (B), SG (C) and SG (D). In addition, the wet weight of SG (E) was significantly lower than SG (B) (two sided t-test, t = 5.244, n = 6, *P* = 0.034) and SG (D) (two sided t-test, t = 11.608, n = 6, *P* < 0.001).

**Figure 2:**
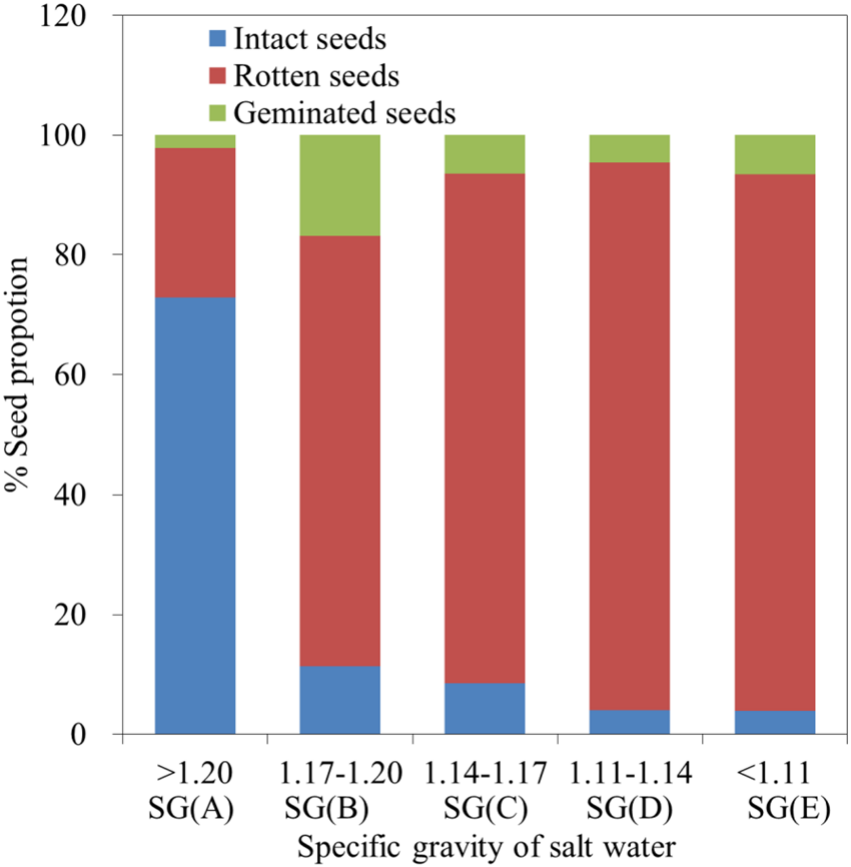
The proportion of geminated, rotten, and intact eelgrass seeds at different specific gravities (SG). The highest proportion of intact seeds (72.91 ± 0.50%) among all five SG classes was recorded for SG (A), which was significantly higher than that of other classes [SG (B–E)] (two sided t-test, *P* < 0.001 for each SG).The vast majority of seeds at classes SG (B–E) were rotten (71.83–91.46%). The viability of intact seeds at SG (A) was tested, and 81.33 ± 4.04% germinated.

Seeds were classified as one of three types (geminated, rotten, or intact) (Fig. 2). The proportion of intact seeds differed significantly (Kruskal-Wallis test, χ2 = 12.833, n = 15, *P* = 0.012) at different SGs (Fig. 2). The highest proportion of intact seeds (72.91 ± 0.50%) among all five SG classes was recorded for SG (A) (*P* < 0.001), which was significantly higher than that of other classes [SG (B–E)] (two sided t-test, *P* < 0.001 for each SG). The proportion of rotten seeds at SG (A) was 24.91 ± 0.15%, which was significantly lower than that of other classes [SG (B–E)] (two sided t-test, *P* < 0.001 for each SG). However, the vast majority of seeds at classes SG (B–E) were rotten (71.83–91.46%). The viability of intact seeds at SG (A) was tested, and 81.33 ± 4.04% germinated. SG (A) accounted for a large proportion (61.93%) of the total wet weight of seeds, but also had the highest proportion of intact seeds. In addition, the intact seeds at SG (A) had high viability, therefore, it appeared that an SG greater than 1.20 is a useful parameter for *Z. marina* seed selection.

### Seed storage with different antibacterial agents

**Figure 3:**
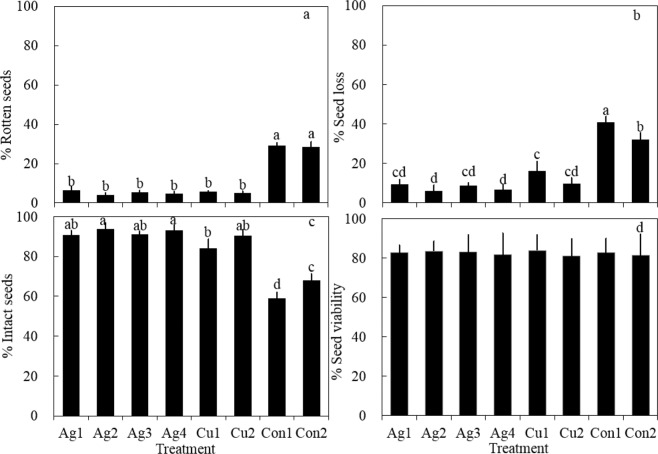
The percentage of rotten seeds (**a**), total seed loss (**b**), intact seeds (**c**), and intact seed viability (**d**) after storage under different conditions for eelgrass. Ag1 and Ag2 represent nano-silver treatments at 2 ppm and 20 ppm, respectively, with a salinity of 32.6 psu. Ag3 and Ag4 represent nano-silver treatments at 2 ppm and 20 ppm, respectively, with a salinity of 50 psu. Cu1 and Cu2 represent copper sulfate treatments (2 ppm) with a salinity of 32.6 psu and 50.0 psu, respectively. Experimental controls (Con1 and Con2) had a salinity of 32.6 psu and 50 psu, respectively, with no added antibacterial agents. RP in the nano-silver (Ag1-4) and copper sulfate (Cu1-2) treatments were significantly lower than those in the control treatments (Con1-2) (two sided t-test, *P* < 0.05 for each treatment). Seed losses in the nano-silver (Ag1-4) and copper sulphate treatments (Cu1-2) were significantly lower than those in the control treatments (Con1-2) (two sided t-test, *P* < 0.05 for each treatment). The lowest seed loss (6.21 ± 3.05%) among all storage conditions was recorded in the nano-silver treatment of 20 ppm with a salinity of 32.6 psu (Ag2). IP in Ag1-4 and Cu1, Cu2 were significantly higher than those of the control treatments (two sided t-test, *P* < 0.05 for each treatment). Over 90% of seeds were intact in the nano-silver treatments, significantly higher than that of control treatments (two sided t-test, *P* < 0.05 for each treatment). The highest percentage of intact seeds (93.79 ± 3.05%) was recorded in Ag2. The viability of intact seeds ranged from 78.00% to 85.33%. Bars represent sd (n = 3).

RP differed significantly (Kruskal-Wallis test, χ2 = 16.942, n = 15, *P* = 0.018) under different storage conditions (Fig. 3a). There were no significant differences in RP between the nano-silver and copper sulfate treatments (two sided t-test, *P* > 0.05 for each treatment). RP in the nano-silver (Ag1-4) and copper sulfate (Cu1-2) treatments were significantly lower than those in the control treatments (Con1-2) (two sided t-test, *P* < 0.05 for each treatment). There were no significant differences in RP between the two control treatments (two sided t-test, n = 6, *P* > 0.05).

Seed losses during storage included geminated and rotten seeds. Seed losses were significantly different (Kruskal-Wallis test, χ2 = 17.938, n = 15, *P* = 0.012) under different storage conditions (Fig. 3b). There were no significant differences in seed losses between the nano-silver treatments (Ag1-4) and the copper sulphate treatment with a salinity of 50 psu (Cu2) (two sided t-test, *P* > 0.05 for each treatment). Seed losses in the nano-silver (Ag1-4) and copper sulphate treatments (Cu1-2) were significantly lower than those in the control treatments (Con1-2) (two sided t-test, *P* < 0.05 for each treatment). The lowest seed loss (6.21 ± 3.05%) among all storage conditions was recorded in the nano-silver treatment of 20 ppm with a salinity of 32.6 psu (Ag2). For control treatments, seed loss at 32.6 psu (Con1) were significantly higher than those at 50 psu (Con2) (two sided t-test, t = 3.486, n = 6, *P* = 0.025).

IP different significantly (Kruskal-Wallis test, χ2 = 17.938, n = 15, *P* = 0.012) between different storage conditions (Fig. 3c). IP in Ag1-4 and Cu1, Cu2 were significantly higher than those of the control treatments (two sided t-test, *P* < 0.05 for each treatment). Over 90% of seeds were intact in the nano-silver treatments, significantly higher than that of control treatments (two sided t-test, *P* < 0.05 for each treatment), while less than 70% of seeds were intact in the control treatments. The highest percentage of intact seeds (93.79 ± 3.05%) was recorded in Ag2. Also, over 80% of seeds were intact in Cu1, Cu2. IP in Control 1 was significantly lower than that in Control 2 (two sided t-test, *P* < 0.05 for each treatment). The results showed that the addition of nano-silver and copper antibacterial agents could significantly reduce rotten seed and seed loss percentage, thus increased intact seed percentage.

The viability of intact seeds was assessed after storage under different conditions. There were no significant differences in GP between the different storage conditions, and seed germination ranged from 78.00% to 85.33% (Fig. 3d).

## Discussion

Selecting seeds using salt water is an ingenious method first used by farmers in ancient China. It mainly utilizes the buoyancy of salt water, which is higher than fresh water, to remove grass seeds, impurities and empty seeds. This method improves seed quality, increases the level of germination, and reduce disease levels. This method has been applied to terrestrial plants including vegetables, crops such as rice^39,41,42,49^, Chinese cabbage^38^, rape^37^, and kohlrabi^43^. However, until now little was known about its application for marine plants, specifically eelgrass seeds. In the present study, it was found that an SG greater than 1.20 resulted in a high proportion of intact seeds (72.91 ± 0.50%) with high seed viability (>80%). The approach can be used as a highly efficient step to select high-quality eelgrass seeds.

Planting seeds is an important method of eelgrass restoration. Eelgrass generally produces mature reproductive shoots in summer, and seeds are buried in the sediment forming a seed bank. The timing of seed germination differs between populations. For example, it was found that there are differences in the timing of seed germination between Huiquan Bay and Swan Lake populations^50^. Seeds in the Huiquan Bay population germinate in the autumn, while those in Swan Lake germinate in spring. Therefore, seeds collected from Swan Lake could to be stored for planting in the spring of the following year. The storage of eelgrass seeds is an essential part of eelgrass population restoration. Generally, seagrass seeds are stored in natural seawater at 4–7 °C^51–54^. Recently, Gu *et al*.^48^ suggested that 0 °C is an appropriate temperature for the long-term storage of *Ruppia sinensis* seeds. Many studies have showed that high salinity levels may inhibit seagrass seed germination^48,51,52,54–57^. Our results (Fig. 3) were consistent with previous studies on salinity; in the control treatments (Con1-2), there was a higher GP at 32.6 psu (Con1; 14.45 ± 3.56%) than at 50 psu (Con2; 3.51 ± 0.93%), indicating that high salinity conditions could reduce seed germination. Also, our previous research indicated that 0 °C and salinity 40–50 psu were suitable conditions for eelgrass seed storage (unpublished data); however, this method still requires regular water changes to reduce microorganism contamination, which increases labor and associated costs. In addition, temperature changes associated with regular water changes may affect seed storage. Given these drawbacks, antibacterial agents were investigated to inhibit the survival and growth of microorganisms, without regular water changes.

Using these antibacterial agents resulted in a low level of seed loss during storage (<10%), and high levels of seed viability (>80%) of the stored intact seeds after 6 months storage at 0 °C. This study revealed that both nano-silver and copper sulfate materials were suitable antibacterial agents for eelgrass seed storage. Silver antibacterial materials have a long application history and have good bactericidal effects on bacteria, viruses and eukaryotic microorganisms. Nano-silver material has a large specific surface area and has superior performances when compared with conventional antibacterial agent^58^. Silver has stronger antibacterial metal bactericidal ability than copper^59–61^. In the present study, the RP in the nano-silver and copper sulfate treatments was lower than 10%, indicating that the nano-silver and copper sulfate agent could reduce microorganism contamination during eelgrass seed storage. Copper has been used as a fungicide since the 19th century^62^. Govers *et al*.^63^ found that copper sulfate under seawater condition is an effective fungicide for the fungi *Phytophthora* and *Halophytophthor*. The present study showed that nano-silver or copper sulfate material can be used as highly effective antibacterial agents in eelgrass seed long-term storage.

It is known that nano-silver has certain toxicity and antibacterial properties, which inhibits the growth of bacteria, algae, prokaryotes, invertebrates and fish in the water, and has a threat to human safety^64^. Thus, seed treatment by nano-silver is recommended only during storage for restoration purposes and not in a natural population in the field.

Accordingly, we proposed some recommendations for seagrass bed restoration using seeds. Before planting or preserving eelgrass seeds, an SG greater than 1.20 can be used as a highly efficient means of selecting high-quality eelgrass seeds. For eelgrass long-term seed storage, it is suggested that seeds should be stored at 0 °C in high-salinity (50 psu) seawater, and nano-silver or copper sulfate (2 ppm) be added to inhibit microorganism infections and seed decay.

## Conclusions

By using different SGs of salt water, high-quality eelgrass seeds were obtained from reproductive shoots. Controlling temperature conditions and adding antibacterial agents during seed storage meant that *Z. marina* seeds could be stored for a long term, at least until planting the following year. This method produced low seed loss levels and high seed viability levels. These findings reveal a highly efficient seed selection method and describe optimal conditions for storing *Z. marina* seeds, and provide a useful reference for the establishment of *Z. marina* seed banks. The findings may also serve as a useful reference for the storage of other threatened seagrass species and facilitate their *ex situ* conservation and habitat restoration.

## Materials and Methods

### Seed collection

*Z. marina* reproductive shoots were collected in July 2018 from Swan Lake, which is in the northeast of Rongcheng city, northern China (37°20′58.7″N, 122°34′26.9″E). Spathes were picked from reproductive shoots, and stored in a 600 μm mesh bag, which was suspended in the marine lagoon until the shoots degenerated and seeds were released. The mesh bag, containing reproductive shoots, was stirred by hand every week. Once released, seeds were collected by sieving in seawater. In the laboratory, the seeds were placed in a circular, aerated flow-through tank (1.2 m × 1.2 m × 1.2 m) until experiments were initiated.

### Seed selection using different specific gravities of salt water

Experiments were initiated in November 2018. A random sample of eelgrass seeds were divided into five classes by floating, using salt (NaCl) solutions with different specific gravities (SGs) as follows: SG (A), greater than 1.20; SG (B), 1.17–1.20; SG (C), 1.14–1.17; SG (D), 1.11–1.14 and SG (E), less than 1.11.

This experiment involved three replicate flasks (2 L), each containing ~100 g (95–105 g) seeds. Wet weight of seeds in each of the five classes were measured. Seed numbers in each of the five classes were counted and the proportion of seeds in each class was calculated. Within each class, each seed was classified as one of three types (geminated, rotten, or intact). The number of seeds within each seed type was counted. Germinated seeds were defined by the emergence and growth of a cotyledon, not just the rupture of the seed coat^65,66^. To distinguish rotten and intact seeds, seeds were gently pressed using plastic tweezers. Rotten seeds were flattened by this process. The percentage of germinated seeds (GP), rotten seeds (RP), and intact seeds (IP) were calculated, to evaluate seed quality from different SG classes. The GP, RP, and IP of the *Z. marina* seeds were calculated using the following equations:

1 $$GP\,( \% )=\frac{g}{N}\times 100 \% ;$$

2 $$RP( \% )=\frac{r}{N}\times 100 \% ;$$

3 $$IP\,( \% )=\frac{i}{N}\times 100 \% ;$$

*g* = the number of germinated seeds, *N* = the total number of seeds, *r* = the number of rotten seeds, and *i* = the number of intact seeds.

### Seed viability test

The viability of intact seeds was tested at a salinity of 10 psu and 15 °C^67^. Seed viability was assessed by germination tests, which involved three replicate flasks (1 L) containing 100 seeds each. The artificial seawater was changed every week and the number of germinated seeds in each flask were recorded. Seed viability tests were carried out for four weeks. The final GP at the end of the experimental period was calculated using Eq. (1).

### Seed storage with different antibacterial agents

The storage experiment was initiated in November 2018. Seeds were rinsed in salt water with an SG of 1.20, with submerged seeds selected for further processing. Intact seeds were selected from submerged seeds. Intact seeds were placed in 1 L jars containing 1 L of sterilized natural seawater (32.6 psu) or artificial seawater (50 psu). Nano-silver (4 nm in diameter, Weijing Nano Corporation; two experimental concentration levels in water: 2 ppm and 20 ppm) and copper sulfate (CuSO_4_·5H_2_O, AR; experimental concentration 2 ppm, according to Govers *et al*.^63^) were added (Table 1). In addition, two treatments, with no added solvent, were established as control groups. The concentration of solvent and the salinity of different treatments are listed (Table 1). Each treatment was replicated in three jars containing ~10 g seeds each. To avoid water evaporation from jars, a layer of plastic wrap was placed on the cap. All jars were stored in the dark at 0 °C. After six months of storage, the storage experiment was terminated and the seeds were retrieved. The stored seeds were classified as one of three types (geminated, rotten, or intact), and the number of each type from each group was counted. Geminated and rotten seeds were regarded as seed losses during storage. The RP and IP were calculated using Eqs. (2, 3). IP was calculated to evaluate the effectiveness of the seed storage methods with different antibacterial agents. In addition, the viability of the stored intact seeds was tested.

**Table 1:** Eelgrass seed storage under different conditions with antibacterial agents.

Treatment	Seawater salinity (psu)	Antibacterial agent	Antibacterial agent concentration in seawater (ppm)
Ag1	32.6	Nano-silver	2
Ag2	32.6	Nano-silver	20
Ag3	50	Nano-silver	2
Ag4	50	Nano-silver	20
Cu1	32.6	Copper sulfate	2
Cu2	50	Copper sulfate	2
Control 1	32.6	None	—
Control 2	50	None	—

*Seawater salinity: 32.6 psu and 50 psu represent the salinity of sterilized natural seawater and artificial seawater, respectively.

### Data and analysis

Because the homogeneity of variance has no significant difference, differences in wet weights of eelgrass seeds selected at different SGs were statistically analyzed using Kruskal-Wallis test. Because the distribution of the data is not normal, differences in the proportion of intact and rotten eelgrass seeds at different SGs were statistically analyzed using Kruskal-Wallis test, and differences in RP, seed loss (RP + GP) and IP of eelgrass seeds after stored at different conditions were also statistically analyzed using Kruskal-Wallis test. Two sided t-test was used to identify specific treatment differences.

For the analysis, homogeneity of variance was tested using Levene’s test^68^. Normality of data was tested using Kolmogorov-Smirnov test and Shapiro-Wilk test. Analysis was carried out on data collected during this study. SPSS 18.0 for Windows 8.1 was used for all data analyses. Differences were considered significant at a probability level of p < 0.05.
